# Comparison of mortality in people with type 2 diabetes between different ethnic groups: Systematic review and meta-analysis of longitudinal studies

**DOI:** 10.1371/journal.pone.0314318

**Published:** 2025-01-17

**Authors:** Umar Ahmed Riaz Chaudhry, Rebecca Fortescue, Liza Bowen, Stephen J. Woolford, Felicity Knights, Derek G. Cook, Tess Harris, Julia Critchley

**Affiliations:** 1 Population Health Research Institute, St George’s, University of London, London, United Kingdom; 2 The Migrant Health Research Group, Institute for Infection and Immunity, St George’s University of London, London, United Kingdom; Naresuan University, THAILAND

## Abstract

**Aims:**

Type 2 diabetes (T2D) is more common in certain ethnic groups. This systematic review compares mortality risk between people with T2D from different ethnic groups and includes recent larger studies.

**Methods:**

We searched nine databases using PRISMA guidelines (PROSPERO CRD42022372542). We included community-based prospective studies among adults with T2D from at least two different ethnicities. Two independent reviewers undertook screening, data extraction and quality assessment using the Newcastle-Ottawa Scale. The primary outcome compared all-cause mortality rates between ethnic groups (hazard ratio (HR) with 95% confidence intervals).

**Results:**

From 30,825 searched records, we included 13 studies (7 meta-analysed), incorporating 573,173 T2D participants; 12 were good quality. Mortality risk was lower amongst people with T2D from South Asian [HR 0.68 (0.65–0.72)], Black [HR 0.82 (0.77–0.87)] and Chinese [HR 0.57 (0.46–0.70)] ethnicity compared to people of White ethnicity. Narrative synthesis corroborated these findings but demonstrated that people of indigenous Māori ethnicity had greater mortality risk compared to European ethnicity.

**Conclusions:**

People with T2D of South Asian, Black and Chinese ethnicity have lower all-cause mortality risk than White ethnicity, with Māori ethnicity having higher mortality risk. Factors explaining mortality differences require further study, including understanding complication risk by ethnicity, to improve diabetes outcomes.

## Introduction

Type 2 diabetes (T2D) is a leading cause of morbidity and mortality worldwide [1]. Compared to people without diabetes, T2D almost doubles the risk of mortality [2], primarily from circulatory conditions, but increasingly so from cancer or neurodegenerative causes [3].

Ethnicity is a widely recognised risk factor for T2D; for instance, people of South Asian and Black ethnicity compared to White ethnicity have a higher prevalence (UK-based crude percentage prevalence by South Asian, Black and White ethnicity: 7.7%, 5.6%, 5.0% respectively), increased incidence (2–3 times) and lower diagnostic age (up to 10 years younger) [4–8]. Ethnicity also influences the subsequent sequelae in terms of developing diabetes-related complications, therefore understanding complication differences by ethnicity is important as it enables consideration of those that could influence mortality risk [4].

Substantial changes in mortality associated with T2D have occurred recently [3, 9]. Vascular-related clinical outcomes have fallen due to implementation of preventative measures, necessitating a review of up to date evidence on ethnic differences [3, 9]. All-cause mortality in diabetes has reduced overall, and this is thought to be due to improved treatment pathways, management of risk factors, and overall lifestyle behaviours [3, 9]. While earlier reviews have highlighted differences in mortality outcomes between ethnic groups in T2D, these differences were not quantified through meta-analysis [10, 11]. Several large cohort studies from different countries, comparing mortality risk between different ethnic groups have been published recently [7, 12–15]. However, a recent systematic review and meta-analysis did not include some of these larger recent cohort studies [7, 12, 15, 16]. It included some historical cohorts (pre-2000) when T2D management was substantially different, cohorts of people with T2D and other health conditions (e.g., those with multiple chronic conditions such as cardiovascular disease, Alzheimer’s disease or stroke), so may not be representative of people with T2D from each ethnicity [16]. The review also did not compare South Asian ethnicity [16] with other ethnicities, and more widely, there have been no previous mortality quantitative comparisons of this group with other ethnicities. A population-wide approach involving all people with type 2 diabetes managed in the community, including those of South Asian ethnicity, would enable strong comparisons between different ethnic groups.

Therefore, a more contemporary and robust analyses of all-cause mortality risk differences in T2D between ethnic groups is required. This is important as it would have key research and clinical implications, especially in driving further research into factors that could explain any mortality risk differences, informing any future focussed clinical interventions and striving towards improved outcomes in T2D. The aim of this systematic review and meta-analysis is to compare mortality risk between people with T2D from different ethnic groups using data from population-based studies.

## Materials and methods

We conducted and reported this systematic review in accordance with the Preferred Reporting Items for Systematic Reviews and Meta-Analyses (PRISMA) guidelines [S1 File] [17]. We prospectively registered our protocol on the international Prospective Register of Systematic Reviews (PROSPERO), CRD42022372542 [18].

### Search strategy

We searched articles in the following nine databases in March 2023, and updated in May 2024: Ovid Medline, Ovid Embase, Ovid PsycInfo, Global Health, Web of Science (Advanced), Cochrane Library, Scopus CINAHL (Plus) and ProQuest (Health Research Premium Collection). The search for Ovid Medline is available in S2 File and was translated to the other databases using the Polyglot Search Translator [19] with some databases being validated through a manual search strategy to ensure correct identification of articles. Studies were identified using a combination of controlled vocabulary with Medical Subject Headings (MeSH) and keyword searching, combined using Boolean logical operators. We finalised search strings for T2D, ethnicity, cohort studies and adult humans through research group consensus. We selected studies published on or after the 1^st^ January 2000, as the management of T2D has significantly evolved over the past two decades. We excluded studies with data collection and follow-up periods ending before 2000 for this reason. To avoid biases, we placed no restrictions on geographical location or language of publication. We selected additional studies after reviewing references from eligible studies, grey literature and earlier systematic reviews.

### Study selection and inclusion/ exclusion criteria

We developed the study inclusion and exclusion criteria using the PICOS framework, and this is outlined in S2 File. Briefly, the population eligible was adults aged ≥18 years with T2D in a population-based or community setting, i.e., cohorts from a general population, primary care or registry-based setting. We placed no restrictions on age, sex, ethnicity, or prior health status in the selected population. Type of diabetes is not always well recorded in larger studies based on electronic health records, and we included studies which did not clearly state diabetes type if we anticipated that the population with T1D was likely very small. We excluded studies incorporating children aged <18 years as these populations are likely to include many patients with T1D, or those focused on type 1 diabetes or gestational diabetes. We also excluded studies if the study population was selected based on a specific co-morbidity (for instance, type 2 diabetes patients with known concomitant chronic kidney disease or cardiovascular disease), since the management of such conditions would be different and could influence future diabetes outcomes.

To be eligible, we required studies to include at least two distinct ethnicity groups to enable comparisons between ethnic groups. We included studies comparing any two ethnic groups, though we anticipated that most studies would have White ethnicity populations as the comparator group. We acknowledge that there are limitations in determining and amalgamating ethnicity groups [20], especially with broad ethnicity categories (for example, South Asian ethnicity incorporates individuals from Bangladeshi, Indian and Pakistani countries of origin); where possible, we used the individual study authors’ ascribed ethnicity groups for data synthesis.

The primary outcome for this systematic review compared all-cause mortality rates between ethnic groups using hazard ratios (HR). We initially included studies reporting all complications of diabetes to ensure that we did not miss mortality outcomes within these studies. For this review, only studies reporting the primary outcome pre-specified in our protocol [18], overall mortality, were included in the analyses.

We included all longitudinal follow-up studies, both prospective and retrospective cohort studies. We also included secondary cohort analyses of randomised controlled trials (RCTs). We excluded other study types, such as RCTs, case series, case control studies, systematic reviews/ meta-analyses, and cross-sectional studies. Where cohorts were duplicated in different publications, we selected the most recent or comprehensive article for inclusion.

We indexed articles retrieved from database searching initially using EndNote X9.3.3 [21], at which point we removed duplicates. Using Rayyan [22], two reviewers independently assessed titles/ abstracts for full-text screening using the inclusion/ exclusion list [S2 File]. Reasons for study exclusion have been provided in supporting information [S3 File]. We obtained the full-texts of records which were again screened [22] by two reviewers independently before inclusion. The two reviewers agreed on and recorded the reason for exclusion at the full-text screening stage. We resolved discrepancies with study selection through discussion between the two reviewers followed by author group consensus if required.

### Data collection

We used a pre-piloted Microsoft Excel data extraction sheet to extract data on study characteristics, which was verified by a second reviewer. We dual extracted outcome data. UC, TH, SW, LB, RF undertook data extraction for all included studies as described in Table 1 from 17^th^ August 2023 onwards. We extracted the following data for each eligible study: 1) study details: first author, year of publication, journal, institutional name of first author, country of institution, funding details; 2) study design: study type (e.g., cohort), data collection period, follow-up period, healthcare setting, country of cohort, total number of participants, follow-up duration, number of ethnicity cohorts, statistical analyses; 3) participants: age minimum, age maximum, mean or median age, sex (percentage female), ethnicity (and percentage of entire cohort), inclusion criteria, exclusion criteria, type 2 diabetes definitions; 4) relevant clinical details and risk factors, for instance type 2 diabetes duration; 5) outcomes: primary outcome comparing all-cause mortality rates; adjustments in outcome reporting based on baseline characteristics and level of adjustment; data format of outcome reporting as HR and 95%CI or other; number of participants in comparative ethnicity groups; any confounding factors; study results. We planned to contact the corresponding author from individual studies if we identified any missing data, but this was not required.

**Table 1:** Summary of study characteristics (13 studies).

Author, year	Country of cohort	Total sample size	Follow-up duration	Comparative ethnicity groups	Characteristics of ethnic groups					
					*N*	Mean age, years (SD)*^†^	Female, %	BMI mean, kg/m^2^	Ever smoked, %^‡^	Diabetes duration, years (SD)
Alharbi et al., 2015 [12]^a^	Australia	12,466	Mean 9.6–11.8 years	Anglo-Celtic	3,608	62.5 (12.0)*	42.2%	33.3	43.7%	NS
				Indigenous Australian	345	52.7 (12.3)*	56.5%	32.3	65.1%	NS
				Pacific Islanders	227	55.0 (11.2)*	49.8%	37.8	44.9%	NS
				Mediterranean	2,217	64.8 (11.0)*	43.6%	31.5	35.2%	NS
				Arabic	493	58.9 (12.0)*	37.9%	33.3	43.0%	NS
				Indian	485	54.0 (12.7)*	37.7%	27.7	29.8%	NS
				Chinese	1,109	62.4 (12.6)*	49.6%	28.5	26.9%	NS
Conway et al., 2015 [13]^a^	USA	12,498	Median 5.9 years	White Americans	3,041	56.2 (9.0)	67.5%	33.9 (median)	63.2%	7.2 (6.9)
				Black Americans	8,978	55.1 (8.9)	66.5%	32.6 (median)	57.2%	7.9 (7.4)
Davis et al., 2010 [28]^b^	Australia	1,057	Mean 9.8 (SD 3.5) years	Anglo-Celt	819	64.8 (11.3)	50.5%	29.6	15.1%^‡^	4.1
				Southern European	238	63.6 (10.3)	54.6%	30.3	12.7%^‡^	5.3
Davis et al., 2014 [27]^b^	UK	4,273	Median 18 years	White Caucasian	3,543	53 (9)	42%	27.9	31%^‡^	NS
				Afro-Caribbean	312	52 (7)	43%	27.1	22%^‡^	NS
				Asian Indian	418	47 (8)	33%	26.0	25%^‡^	NS
Joshy et al., 2010 [31] ^b^	New Zealand	7,501	NS [follow-up duration expected up to 5 years]	European	4,948	57.7 (12.6)^†^	NS	NS	NS	7.8 (6.9)
				Māori	1,749	46.5 (12.4)^†^	NS	NS	NS	8.8 (7.9)
Khan et al., 2011 [14]^a^	Canada	276,837	Median 4.0 years	White	244,017	61.3 (13.1)^†^	45.1%	NS	NS	NS
				Chinese	17,754	59.7 (12.8)^†^	47.6%	NS	NS	NS
				South Asian	15,066	56.5 (12.3)^†^	44.3%	NS	NS	NS
Lee et al., 2018 [29]^b^	USA	19,905	NS [follow-up duration expected up to 12 years]	White	18,522	63.1	45.7%	31.3	16.4%^‡^	NS
				Asian (all)	1,383	59.7	46.3%	27.0	11.9%^‡^	NS
Liu et al., 2018 [30]^b^	Singapore	2,061	Mean 5.5 (SD 2.9) years	Chinese	1,302	58.3 (12.5)	37.7%	26.2	12.5%^‡^	11.9 (9.0)
				Malay	403	56.9 (11.0)	44.5%	28.9	16.3%^‡^	9.8 (7.7)
				Asian Indian	356	55.0 (11.0)	43.3%	27.0	13.1%^‡^	11.4 (8.2)
Lynch et al., 2010 [32]^a^	USA	8,812	Mean 4.5 years	Non-Hispanic White	5,666	63.1 (10.6)	2%	NS	NS	NS
				Non-Hispanic Black	3,146	58.5 (12.0)	3%	NS	NS	NS
Mathur et al., 2018 [15]^a^	UK	6,274	Mean 9.0 years	White	2,447	69.5 (9.3)	58.1%	NS	16.2%^‡^	7.8 (6.7)
				South Asian	2,732	65.5 (9.9)	51.6%	NS	12.9%^‡^	9.9 (7.7)
				Black	1,095	67.8 (10.0)	57.5%	NS	7.0%^‡^	10.8 (8.3)
McEwan et al., 2012 [33]^a^	USA	8,334	Mean 6.2 years	Non-Hispanic White	3,418	NS	NS	NS	NS	NS
				Hispanic	1,371	NS	NS	NS	NS	NS
				African American	1,421	NS	NS	NS	NS	NS
				Asian/Pacific Islander	1,376	NS	NS	NS	NS	NS
				Other	748	NS	NS	NS	NS	NS
Wright et al., 2017 [7]^a^	UK	187,968	Mean 5.0 (SD 3.8) years	White	143,724	63.3 (13.9)	45.3%	31.6	30.0%^‡^	NS
				South Asian	9,523	52.5 (13.6)	45.8%	28.8	26.0%^‡^	NS
				Black	4,461	53.9 (13.9)	50.5%	31.1	22.4%^‡^	NS
Yu et al., 2021 [34]^b^	New Zealand	45,072	Median 9.7 (IQR 5.8–13.6) years	European	16,755	62.2 (13.2)	45.1%	31.2	10.8%^‡^	4.7 (1.2)
				Māori	7,093	51.5 (12.6)	50.9%	35.9	30.0%^‡^	5.0 (1.4)
				Pacific	12,044	52.8 (12.8)	53.5%	35.0	14.4%^‡^	4.9 (1.1)

The ethnicity stated at the top of each group is the nominated comparator. Ethnicities stated are as defined in individual studies.

NS–not stated. SD–standard deviation. IQR–interquartile range.

^a^ denotes studies included in meta-analysis.

^b^ denotes studies presented on a forest plot and / or synthesised narratively.

*N* provides number of patients stratified by ethnic groups with available demographic and clinical data.

Mean age * at last visit or ^†^ at diagnosis, if available. ^‡^ provides % for current smoker if ever smoking data not available.

### Quality assessment

We assessed the methodological quality (risk of bias) of cohort studies in meta-analyses using the Newcastle-Ottawa Scale (NOS), to appraise all included studies within this systematic review [23]. This is a check-list of eight items structured under three sub-headings (selection, comparability, outcomes) with a final rating as either good, fair or poor according to Agency for Healthcare Research and Quality (AHRQ) standards [23]. Two reviewers independently assessed the quality of included studies using the NOS, with any disagreements resolved through discussion.

### Data analysis

As previously outlined, the summary estimate was comparative all-cause mortality rates between ethnic groups. We anticipated that data would be reported as a hazard ratio (HR) with 95% confidence intervals (95%CI). Two reviewers independently verified outcome data and, where necessary, consulted a third reviewer to assess eligibility for inclusion in the meta-analysis.

We undertook meta-analyses where all-cause mortality risk comparisons between ethnic groups were reported as HRs in two or more studies. If results were reported using other statistical measures (not HRs), we included the study in a narrative synthesis. Due to the likelihood of between-study heterogeneity, we used the random-effects inverse-variance model and created Forest plots using RevMan 5.4 [24, 25]. We planned to assess publication bias using funnel plots if a sufficient number of studies (≥10 studies) could be pooled for meta-analyses. We used the I^2^ statistic to assess statistical heterogeneity [25, 26].

Although we did not specify a reference group for this systematic review, we planned to re-adjust HR estimates if the comparator group was non-White ethnicity to enable similar relative comparisons between studies. When data were provided according to different sexes, we added the effect estimate for each sex into the analysis separately rather than attempting to pool the results.

Finally, where individual studies provided more than one statistical model based on adjustment for different numbers of risk factors, then we preferred meta-analysis based on minimally adjusted models (adjusting for only age and sex). This is because maximally adjusted models may be over-adjusted, including variables that may be diabetes complications. Where minimally adjusted models were not available, we presented maximally adjusted models, and where possible, those studies that provided their maximally (i.e., fully-) adjusted models were then compared with their minimally adjusted models. The precise categorisation of ethnicity by study authors and the adjustment factors were provided as footnotes on Forest plots.

### Changes from original protocol

We only undertook meta-analysis where we considered the study design, population, quality and outcomes to be sufficiently similar for pooling to make sense. For this reason, we included Davis *et al*’s (2014) study only in the narrative synthesis [27].

## Results

### Study selection

**Fig 1:**
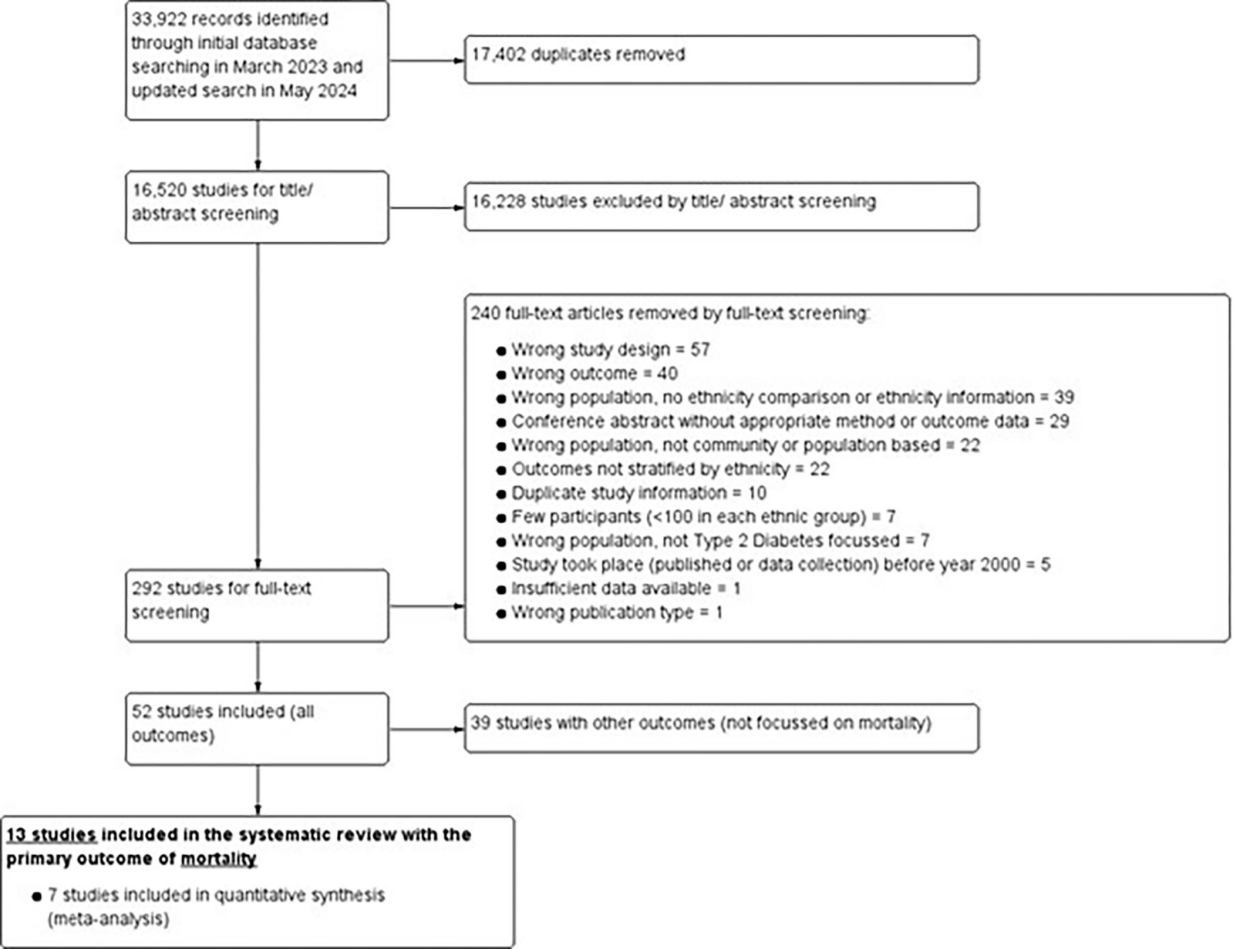
PRISMA flow diagram of literature search, eligibility and study selection.

The PRISMA flow diagram in Fig 1 describes the study selection following the inclusion/ exclusion criteria as described in the Methods section. We retrieved 33,922 studies in total from searching nine databases initially in March 2023 and updated in May 2024, which included a further 3,097 studies. After removing duplicates, 16,520 studies underwent title/ abstract screening, resulting in 292 studies for full-text screening. Amongst these, 13 studies included mortality outcomes, the purpose of this paper, and were therefore eligible for inclusion [7, 12–15, 27–34]. Of these 13 studies, 7 studies [7, 12–15, 32, 33] had sufficient relevant data to be included in the meta-analysis, and the remaining studies [27–31, 34] were reported narratively only.

### Study characteristics

Table 1 describes the study details of the 13 included studies, published between years 2010–2021[7, 12–15, 27–34], and provide characteristics data with each ethnic group. Four studies were conducted in USA [13, 29, 32, 33], three were UK-based [7, 15, 27], two each in New Zealand [31, 34] and Australia [12, 28], and one each in Canada [14] and Singapore [30]. The total number of participants with type 2 diabetes in the studies combined was 573,173. As specified in our protocol, all participants were recruited from community settings [7, 12–15, 27–34]. A range of ethnicities were compared within this review, and described further below; White/ European/ Anglo-Celtic ethnicity was the main comparator in 12 out of the 13 studies [7, 12–15, 27–29, 31–34] and one study compared Chinese ethnicity with Malay and Indian ethnicity [30]. Study follow-up periods ranged from 4 to 18 (median) years.

### Risk of bias (quality) assessment

Using the NOS, and described further in S2 File, 12 studies were rated as Good [7, 12–15, 28–34] with a total score ranging between 7–9. Most studies ensured that people enrolled in the registries were alive at the start of the follow-up period, controlled for main confounders and had adequate follow-up. One study was rated as Poor and had a total score of 6 [27], mainly due to inadequate follow-up of cohorts.

### All-cause mortality of South Asian, Black, Chinese, Western Pacific and Māori ethnicity versus White ethnicity (forest plots)

**Fig 2:**
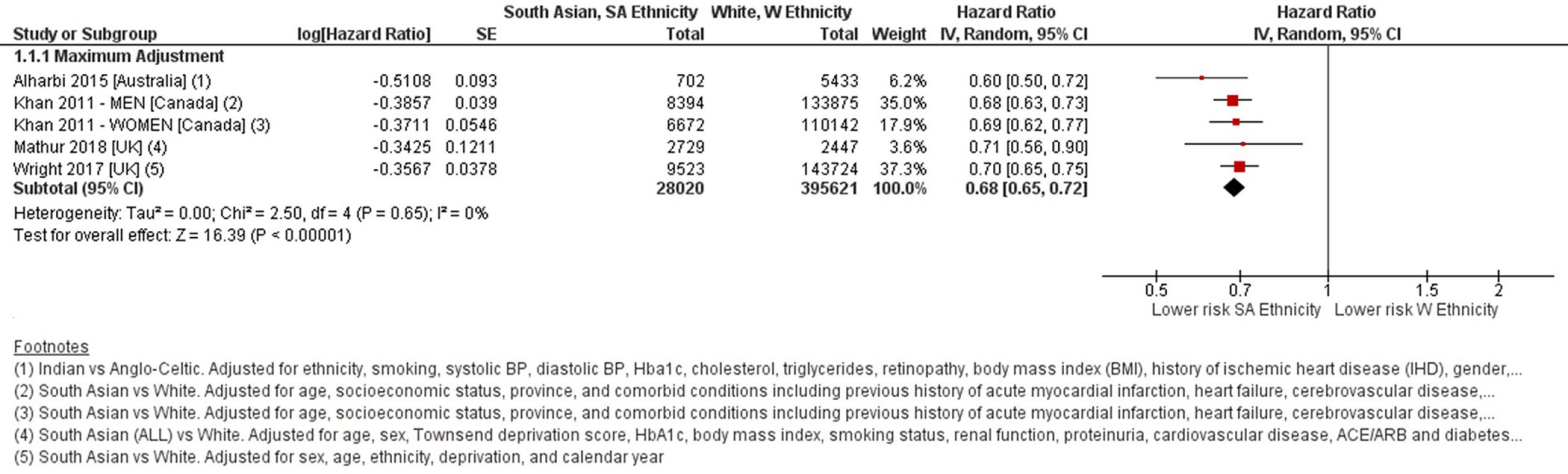
Forest plot comparing all-cause mortality risk between South Asian ethnicity and White ethnicity. Defined ethnic groups within each study in Forest plot footnotes. Adjustment factors also described in footnotes. SA–South Asian ethnicity. W–White ethnicity.

Four studies compared 28,020 people with T2D of South Asian ethnicity to White ethnicity [7, 12, 14, 15]; one stratified analyses by sex [14]. Meta-analysis revealed a lower risk of all-cause mortality among people of South Asian ethnicity compared to White ethnicity, with a HR 0.68 (95%CI 0.65–0.72, p<0.001), I^2^ = 0% [Fig 2].

**Fig 3:**
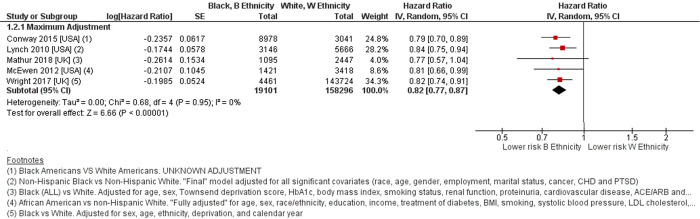
Forest plot comparing all-cause mortality risk between Black ethnicity and White ethnicity. Defined ethnic groups within each study in Forest plot footnotes. Adjustment factors also described in footnotes. B–Black ethnicity. W–White ethnicity.

Meta-analysis of five studies comparing 19,101 people with T2D of Black ethnicity [7, 13, 15, 32, 33] to those of White ethnicity, found a lower risk of all-cause mortality amongst the Black ethnicity group, HR 0.82 (0.77–0.87, p<0.001), I^2^ = 0% [Fig 3].

**Fig 4:**
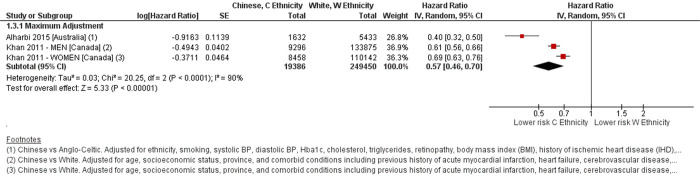
Forest plot comparing all-cause mortality risk between Chinese ethnicity and White ethnicity. Defined ethnic groups within each study in Forest plot footnotes. Adjustment factors also described in footnotes. C–Chinese ethnicity. W–White ethnicity.

Two studies which involved 19,386 people with T2D of Chinese ethnicity compared all-cause mortality risk with people of European ethnicities [12, 14], of which one study separated their analysis by sex [14]. Meta-analysis revealed a lower risk of all-cause mortality amongst the Chinese ethnicity population, HR 0.57 (0.46–0.70, p<0.001) but with substantial heterogeneity, I^2^ = 90% [Fig 4].

Other Western Pacific ethnicities have been compared with White ethnicity in three studies [12, 33, 34]. We did not pool these statistically as it was unclear whether the relevant Western Pacific sub-populations were sufficiently similar. We have therefore presented the effect estimates from the three studies visually in S2 File. Two of the studies demonstrated no difference in all-cause mortality between Western Pacific and White ethnicity [12, 34], whilst the third showed a reduced mortality risk within the Western Pacific ethnic group [33].

The Māori population were directly compared with those of European ethnicity in two studies from New Zealand [34, 35]. Once again, we did not pool these as one study reported adjusted IRRs rather than as a HR [34]. Both studies found a higher risk of all-cause mortality within the Māori ethnicity population, when compared to the White ethnicity population; (HR 1.92 (1.61–2.29) [31] and adjusted IRR 1.96 (1.86–2.13) [34], see S2 File).

### Other ethnic groups versus White ethnicity comparisons (narrative)

We narratively synthesised studies if ethnicity was not clearly described or the reported ethnicity was not consistent with other studies. Alharbi *et al* (2015) examined Mediterranean and Arabic ethnicities in Australia, both of which had a lower risk of mortality compared with Anglo-Celtic ethnicity, with respective HRs of 0.8 (0.7–0.9) and 0.7 (0.6–0.8) [12]. However, people with T2D of Indigenous Australian ethnicity had a higher risk of death compared to White ethnicity, HR 2.3 (1.7–3.0). As part of the Fremantle Diabetes Study also in Australia, Davis *et al* (2010) compared Southern European ethnicity with Anglo-Celtic participants and found no clear difference in their all-cause mortality risk, but with wide confidence intervals (HR 0.96, 0.71–1.31) [28].

Davis *et al*’s (2014) prospective study of 4,273 UK Prospective Diabetes Study participants compared Asian Indian and Afro-Caribbean people with those of White ethnicity [27]. These comparisons were not grouped with the overall quantitative synthesis above due to heterogeneity in quality assessment, study design and outcome reporting. The study reported a lower risk of all-cause mortality amongst those of Asian Indian (RR 0.89, 0.80–0.97) and Afro-Caribbean (RR 0.84, 0.76–0.93) ethnicity, compared to White Caucasian ethnicity in their maximally adjusted model [27]. These findings support those from the meta-analysis for these ethnic groups.

Within the USA, Lee *et al*’s (2018) study used a broad definition of Asian Americans, which encompassed people of “Chinese, Filipino, Asian Indian, and other Asian” backgrounds, and included “American Indian, Alaska Native and individuals of multiple races without a primary race” [29], therefore not possible to compare with other studies focussed only on South Asian ethnicity. Compared to people of White ethnicity, there was a lower risk of all-cause mortality amongst people with T2D of Asian ethnicity in both a model adjusted for age/ sex alone (HR 0.6, 0.4–0.7) and a maximally adjusted model (HR 0.7, 0.5–0.9) [29]. McEwen *et al*’s (2012) study with 8,334 participants, also in the USA and incorporating Hispanic and Other race/ ethnic groups, further demonstrated a lower risk of mortality in these two ethnic groupings compared to their non-Hispanic White ethnicity counterparts (HR 0.78, 0.62–0.97 and HR 0.69, 0.54–0.89 respectively) [33].

In the UK, Mathur *et al*’s (2018) study based in inner London was able to delineate mortality comparisons by ethnicity further, estimating risk amongst Indian, Pakistani and Bangladeshi ethnicities, as well as African and Caribbean ethnicities [15]. Despite identifying lower risk of mortality in Indian and Pakistani ethnicities (HR 0.81, 0.56–1.15 and HR 0.96, 0.59–1.55 respectively), only people of Bangladeshi ethnicity had a lower risk of mortality reaching statistical significance (HR 0.63, 0.46–0.86) when compared to the White ethnicity reference group [15]. African and Caribbean ethnicity participants also had a lower risk of mortality (HR 0.79, 0.43–1.44 and HR 0.77, 0.55–1.08 respectively) [15].

### Other ethnic groups versus non-White ethnicity comparisons (narrative)

Only Liu *et al*’s (2019) study, which was based in Singapore, compared mortality risk with a non-White ethnicity reference group [30]. Here, Chinese ethnicity was the nominated comparator group, and findings suggested a higher risk of mortality within the Malay (HR 1.42, 1.05–1.91) and Asian Indian ethnicity (HR 1.26, 0.86–1.85) populations in their maximally adjusted models [30].

### Minimally versus maximally adjusted models

Some studies provided effect estimates from more than one statistical model based on the number and type of variables for which the effect estimate was adjusted. Only one study, Lynch *et al* (2010), provided sufficient data to enable comparisons between its maximally and minimally adjusted (only for age and sex) as seen in S2 File [32]. Amongst people of Black ethnicity, the race/ age adjusted HR was 0.92 (0.82–1.03) when compared to people of White ethnicity, whereas including all significant co-variates increased the difference between ethnic groups with a HR of 0.84 (0.75–0.94) [32].

## Discussion

### Principal findings

This study demonstrates that people with T2D of South Asian, Black and Chinese ethnicity have a lower risk of all-cause mortality compared to people of White ethnicity. In their respective comparisons to people of White ethnicity, people with T2D of Chinese ethnicity had a 43%, South Asian ethnicity had a 32% and Black ethnicity had an 18% lower relative risk of all-cause mortality. This review also highlights that people from indigenous populations, i.e., Māori New Zealanders and Indigenous Australians, had a higher risk of all-cause mortality compared to people of European/ Anglo-Celtic ethnicity.

### Study strengths and weaknesses

The main strength of this review is that it incorporated 13 population-based studies, involved a total of >500,000 participants, and was carried out according to robust guidelines. Particularly, for comparisons involving people of South Asian and Black ethnic groups, the results were consistent amongst different studies and country populations, and there was very little statistical heterogeneity within these analyses. This is especially noteworthy given different healthcare systems, socio-cultural differences (in diet, lifestyle and approach to clinical management) and levels of healthcare access between countries. Consistency between our findings from meta-analysis and those from the narrative synthesis gives further weight to these conclusions. The review also incorporated a number of recent (published 2010 onwards), large, longitudinal studies focussed on T2D patients only, and our decision to focus on more recent population-based studies of T2D patients only might explain the very low statistical heterogeneity (*I*^*2*^ = 0%) for South Asian and Black ethnicity mortality comparisons with White ethnicity. As far as existing literature is known, this is the first systematic review to provide precise comparative all-cause mortality estimates, especially for people of South Asian ethnicity.

There are a number of limitations of this systematic review. Firstly, the included studies were diverse, incorporating a variety of populations, demographic characteristics, confounders and duration of follow-up periods, which may affect the generalisability of this review. For instance, Lynch *et al* studied the Veterans Health Administration cohort, and it is well-established that veterans in the USA have greater access to healthcare and are different in terms of their socio-demographic composition as compared to the general population [32]. Therefore, despite being a community-based cohort, the findings of these studies may not be fully generalisable [32]. Secondly, we used broader ethnic categories (e.g., South Asian and Black ethnicity) to enable comparisons with people of White ethnicity, as data from further stratified ethnic groups were rarely provided by individual studies. Using broad ethnic categories to identify overarching patterns due to some shared healthcare experiences can sometimes be helpful, however, can also be problematic as it does not fully outline any extent of disparities [36]. By taking an “average” mortality across a broad ethnic grouping, which in itself might not be well-defined, some important variations may be masked within that ethnic group. Thirdly, the included studies used a variety of statistical models to adjust for different co-variates. Most studies included in the meta-analysis were the maximally adjusted models, which possibly risks over-adjustment. Fourthly, some studies were not included in the meta-analysis as they were not compatible with the other studies in the analysis. However, we included these studies in the narrative review and findings were consistent with those from meta-analysis. Fifthly, we were not able to sufficiently assess publication bias as no meta-analysis contained ≥10 studies. Finally, despite not limiting the search by geography, the majority of these studies were conducted in North America (USA and Canada), the UK and Australasia, which brings into question the generalisability of these findings in other countries with different healthcare systems. Only one study had a non-White reference group, which therefore limits comparisons in countries where White ethnicity is not the predominant ethnic category.

### Comparisons with other studies

Previous systematic reviews have explored mortality differences in T2D by ethnicity. Earlier reviews without meta-analysis described higher risk of mortality and complications in people with T2D compared to those without T2D, more generally, alluding to ethnic differences in diabetes care and complications [10, 11]. A more recent systematic review provided T2D mortality risk estimates for people of Black ethnicity compared to White ethnicity, with overall similar estimates to this systematic review (HR 0.93, 0.79–1.10) [16]. Our comparative mortality risk estimate between Black and White ethnicity is further from the null value (1.0), more precise, reaches levels of statistical significance (HR 0.82, 0.77–0.87) and has very low statistical heterogeneity (*I*^*2*^ = 0%) [16]. This earlier systematic review, also did not include some large cohort studies published more recently, which is significant as T2D management has changed substantially over time [16]. This review, Ezzatvar *et al* (2021), included studies selected on the basis of certain co-morbidities alongside T2D, therefore bringing into question its application to a more general T2D population [16]. Furthermore, Ezzatvar *et al* (2021) did not provide any comparative mortality risk estimates between South Asian ethnicity and other ethnicities, which is estimated in our systematic review and important in the context of T2D [16]. Similar to our review, Ezzatvar *et al* (2021) also highlighted an increased mortality risk amongst the indigenous Māori people, compared to people of White ethnicity (HR 1.88, 1.61–2.21) [16].

A number of reasons have been postulated to explain the lower risk of all-cause mortality in South Asian, Black and Chinese ethnicity people with T2D compared to White ethnicity populations [37, 38]. People with T2D amongst these ethnic groups are often younger, and therefore less likely to already have cardiovascular or other health conditions at diagnosis [37], to have an impact on mortality. There is also emerging evidence that diabetes can develop in people of South Asian with lower body mass index, compared to White ethnicity groups [4, 38, 39]. Moreover, it is known that non-White ethnicity groups in the UK are commenced on non-insulin monotherapy sooner than their White ethnicity counterparts, though future intensification to combination therapy and insulin is quicker in White ethnic groups [6].

T2D complications also differ by ethnicity, which could influence future mortality risk. There are differences in risk reported in macrovascular (ischaemic heart disease, cerebrovascular disease and peripheral vascular disease) and microvascular (nephropathy, neuropathy and retinopathy) conditions [4, 5]. For instance, compared to White ethnicity, those from Black/ South Asian ethnicity with T2D have: a higher risk of retinopathy, nephropathy and stroke; an equal or reduced risk of lower limb amputation, peripheral neuropathy and overall cardiovascular complications, but with South Asian populations experiencing higher coronary heart disease rates [5]. Furthermore, alongside fixed risk factors (such as age, sex and genetics), modifiable risk factors (for example, lipid levels, blood pressure, obesity, smoking status, physical activity levels, renal function), clinical management in T2D (for instance, glycated haemoglobin, variation in initiation or intensification of diabetes treatment), healthcare access and socio-economic factors are thought to underpin many of these ethnic differences in diabetes complication risk, including mortality [4–6]. For our overall programme of work on ethnic differences in T2D, all diabetes complications were initially considered for selection [3]. For the purpose of this specific review, we focused on mortality outcomes only. It is anticipated that further systematic reviews will seek to compare these other secondary outcomes in T2D.

The finding of lower mortality and higher life expectancy in UK South Asian and Black ethnicity groups in people with T2D mirrors the ethnic differences in mortality seen in the overall UK population [40]. One possible explanation for this is the ‘healthy migrant effect’, whereby individuals who are healthier are more likely to migrate [40]. Prevalence of some risk behaviours such as alcohol consumption and smoking also tend to be lower in non-White ethnicity groups, and it is important to acknowledge the complex interplay of biological, environmental, socio-economic, behavioural and cultural factors that could also explain these health disparities [40]. Whether these ethnic patterns of mortality are attenuated by other factors such as socio-economic status or healthcare access, requires further analyses. More recently and during the pandemic in the UK, COVID-19-related mortality was higher amongst South Asian and Black ethnicities compared to White ethnicity, though non-COVID-19 related mortality remained higher for those of White ethnicity [41].

Whilst the ‘healthy migrant effect’ could have a positive influence on ethnic groups in certain countries, indigenous populations such as native and other Pacific Islanders populations in the USA and elsewhere are known to be disproportionately affected by T2D by having a higher mortality risk [42]. They often experience significant disadvantage, including economic segregation, poor access to healthcare and more likely to engage in known risk behaviours [43, 44]. This can perpetuate existing health disparities amongst native populations and increase the risk of developing future diabetes-related complications, extending disparities according to the ‘inverse care law’ in T2D [43–45].

### Implications of study findings and future research

This systematic review has shown that in people with T2D, White ethnicity groups were identified to be at higher risk of all-cause mortality compared to South Asian, Black and Chinese ethnic groups. This is demonstrated through both our meta-analysis and narrative synthesis. From a clinical perspective, raising awareness of mortality risk differences could identify populations at highest risk of certain T2D complications, which could help to inform strategies and improve modifiable risk factor profiles. T2D is less common in White ethnicity compared to South Asian and Black ethnicity groups, but is often diagnosed at an older age [4], and therefore individuals are more likely to have more co-morbidities with greater metabolic risk, especially amongst lower socio-economic populations, which could manifest itself as higher risk of serious T2D complications and mortality. Most studies did not report detailed information on socio-economic status; gradients of socio-economic deprivation across different ethnic groups should also be explored to determine the extent to which these might explain mortality risk differences both among people with T2D and in the general population. Comparisons of risk in developing serious macrovascular and microvascular complications by ethnicity, which could lead to differences in mortality risk, also needs further scrutiny, with particular attention to effect modifiers underpinning any health outcome differences in T2D.

This research has also identified significant differences between studies in the methods of adjustment for confounders. Studies in this review mainly provided their maximally adjusted models, which were not directly comparable to other studies, and so we would encourage those conducting future cohort analyses to also provide minimally adjusted models (e.g., by age and sex only) to enable these comparisons. Furthermore, adhering to relevant confounder selection, avoiding over-adjustment and enabling a standardized approach to outcome reporting in T2D by ethnicity would be beneficial in future research [46]. This review also emphasized the importance of future work examining mortality risks between different non-White ethnicity groups. Only one study based in Singapore was included within this review comparing Chinese ethnicity with Malay and Indian ethnicity [30]. Other comparisons of this type would enable more generalised mortality risk patterns by ethnicity, which could be extrapolated across different populations and countries.

Finally, this review has highlighted the need to further scrutinise key risk factors explaining mortality differences by ethnicity in T2D. This would be helpful to determine whether tailored interventions by healthcare professionals are required to manage these risk factors and prevent long-term complications, including mortality. Future work is required to understand factors that could explain mortality differences, including complication risk by ethnicity, to improve diabetes outcomes.

## Supporting information

S1 File Prisma checklist. (DOCX)

S2 File Supplementary tables and figures. (DOCX)

S3 File Reasons for study exclusion. (CSV)
