# Supplementary material for: Comparison of mortality in people with type 2 diabetes between different ethnic groups: Systematic review and meta-analysis of longitudinal studies
Source: PLoS One. 2025 Jan 17;20(1):e0314318. doi: 10.1371/journal.pone.0314318 (PMC11741655; doi:10.1371/journal.pone.0314318)
Supplement: S2 File — (DOCX) [file pone.0314318.s002.docx]

Supplementary Tables and Figures

​​

[Supplementary Material Only 2](#_Toc160106003)

[Table S1 – Ovid Medline Search Strategy 2](#_Toc160106004)

[Table S2 – Inclusion/ Exclusion Criteria for Literature Screening 3](#_Toc160106005)

[Table S3 – Quality assessment using the Newcastle-Ottawa Scale 4](#_Toc160106006)

[Figure S4 – Forest plot comparing all-cause mortality risk between Western Pacific ethnicity and White ethnicity 5](#_Toc160106007)

[Figure S5 – Forest plot comparing all-cause mortality risk between Māori ethnicity and White ethnicity 6](#_Toc160106008)

[Figure S6 – Minimally versus maximally adjusted models within the Black ethnicity comparisons 7](#_Toc160106009)

​

# Supplementary Material Only

## Table S1 – Ovid Medline Search Strategy

Ovid MEDLINE(R) and Epub Ahead of Print, In-Process, In-Data-Review & Other Non-Indexed Citations, Daily and Versions

1 Diabetes Mellitus, Type 2/

2 type 2 diabetes.tw.

3 (MODY or NIDDM or T2D*).tw.

4 (non insulin* depend* or noninsulin* depend* or noninsulin?depend* or non insulin?depend*).tw.

5 1 or 2 or 3 or 4

6 ethnic group/

7 racial groups/

8 group, minority/

9 minority health/

10 population groups/

11 ethnic*.tw.

12 (race or racial).tw.

13 minorit*.tw.

14 (bame or bme).tw.

15 continental population groups/

16 Hispanic Americans/

17 African Continental Ancestry Group/

18 American Native Continental Ancestry Group/

19 asian continental ancestry group/

20 european continental ancestry group/

21 oceanic ancestry group/ 0

22 african americans/

23 arabs/ 5451

24 americans, asian/ 821

25 (multi*cultural or multi cultural or cross*cultural or cross cultural or trans*cultural or transcultural).tw.

26 asians/

27 blacks/

28 whites/

29 6 or 7 or 8 or 9 or 10 or 11 or 12 or 13 or 14 or 15 or 16 or 17 or 18 or 19 or 20 or 21 or 22 or 23 or 24 or 25 or 26 or 27 or 28

30 Cohort Studies/

31 Longitudinal Studies/

32 Follow-up Studies/

33 Prognosis/

34 Risk Factors/

35 cohort*.tw.

36 longitudinal.tw.

37 (follow up or follow-up).tw.

38 prognos*.tw. 768561

39 (risk* adj3 factors*).tw.

40 30 or 31 or 32 or 33 or 34 or 35 or 36 or 37 or 38 or 39

41 5 and 29 and 40

42 Infant/ 860724

43 infant*.ti,ab.

44 Child/

45 child*.ti,ab.

46 42 or 43 or 44 or 45

47 41 not 46

48 limit 47 to (humans and yr="2000 -Current")

## Table S2 – Inclusion/ Exclusion Criteria for Literature Screening

| General Properties | Inclusion | Exclusion |
| --- | --- | --- |
| Participants / Population | Adults ages ≥18 years  People with type 2 diabetes  Population- or community-based – representative of the general population  [No restriction on age, sex, ethnicity or prior health status] | Children aged <18 years  People with type 1 diabetes or gestational diabetes or pre-diabetes  Institutional- or hospital-based  Ethnic group with <100 participants  Selected on the basis of sub-group populations for another health condition |
| Intervention(s) / Exposure(s) | All ethnic groups | Ethnicity-specific populations or populations with a single ethnic group |
| Comparator(s) / Control | At least two different ethnic groups within the entire cohort identified | Not necessary to have White ethnicity as the comparator |
| Outcome(s) | All-cause mortality (primary outcome)  • Cause-specific mortality (e.g. cardiovascular mortality, cancer mortality etc)  • Complications from diabetes will be:  - Macrovascular: cerebrovascular disease (e.g., stroke/ TIA); cardiovascular disease (e.g., IHD, angina, CABG/ PCI); peripheral vascular disease (e.g., angioplasty, stent insertion or amputations) - Microvascular: chronic kidney disease; retinopathy; peripheral neuropathy  - Other conditions: dementia; infections; cancers; mental health  - Hospitalisations (both elective and emergency)  - Other clinical outcomes not specified | • Other modifiable risk factors related to type 2 diabetes: HbA1c, medications, cardiometabolic risk markers (e.g., obesity, circulating cholesterol levels, renal function and blood pressure)  • Outcomes not specified in inclusion criteria |
| Study Design | Longitudinal follow-up studies: prospective and retrospective cohort studies  Secondary analysis from cohort data within RCTs | RCTs; case series; case-control studies; systematic reviews / meta-analysis (except for study identification)  Cross-sectional studies |

## Table S3 – Quality assessment using the Newcastle-Ottawa Scale

| Study # | Author and year of publication | Newcastle-Ottawa Quality Assessment Form for Cohort Studies | | | | |
| --- | --- | --- | --- | --- | --- | --- |
|  |  | Selection | Comparability | Outcome | **Total** | AHRQ standards |
| 1 | Alharbi et al., 2015[8] | 4 | 2 | 3 | **9** | Good |
| 2 | Conway et al., 2015[9] | 4 | 1 | 3 | **8** | Good |
| 3 | Davis et al., 2010[24] | 3 | 1 | 3 | **7** | Good |
| 4 | Davis et al., 2014[25] | 3 | 2 | 1 | **6** | Poor |
| 5 | Joshy et al., 2010[28] | 3 | 1 | 3 | **7** | Good |
| 6 | Khan et al., 2011[10] | 3 | 2 | 4 | **8** | Good |
| 7 | Lee et al., 2018[26] | 3 | 2 | 4 | **8** | Good |
| 8 | Liu et al., 2018[27] | 3 | 2 | 4 | **8** | Good |
| 9 | Lynch et al., 2010[29] | 3 | 2 | 4 | **8** | Good |
| 10 | Mathur et al., 2018[11] | 4 | 1 | 2 | **7** | Good |
| 11 | McEwan et al., 2012[30] | 3 | 1 | 3 | **7** | Good |
| 12 | Wright et al., 2017[7] | 4 | 1 | 3 | **8** | Good |
| 13 | Yu et al., 2021[31] | 3 | 1 | 3 | **7** | Good |

Agency for Healthcare Research and Quality (AHRQ)

##
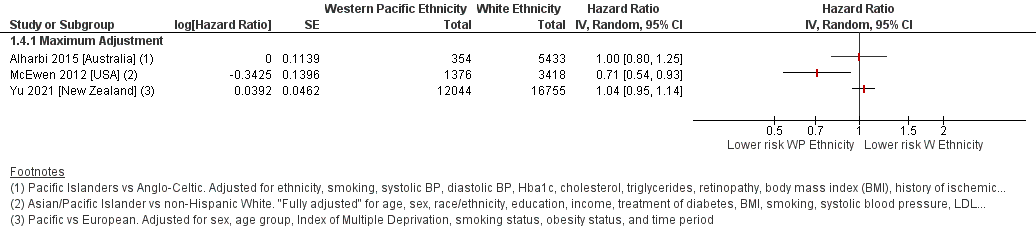
Figure S4 – Forest plot comparing all-cause mortality risk between Western Pacific ethnicity and White ethnicity

Defined ethnic groups within each study in Forest plot footnotes. Adjustment factors also described in footnotes. Yu 2021 reported incident rate ratios (IRRs).

WP – Western Pacific ethnicity. W – White ethnicity.

##
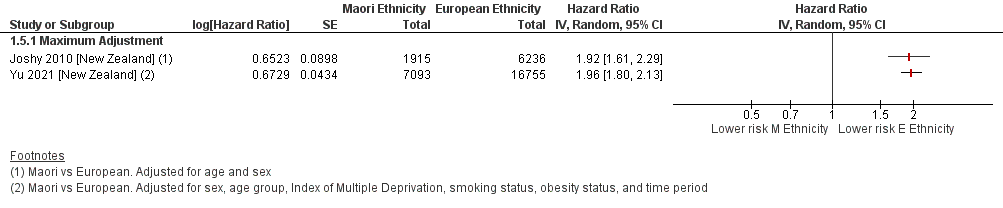
Figure S5 – Forest plot comparing all-cause mortality risk between Māori ethnicity and White ethnicity

Defined ethnic groups within each study in Forest plot footnotes. Adjustment factors also described in footnotes. Yu 2021 reported incident rate ratios (IRRs).

M – Māori ethnicity. W – White ethnicity.

##
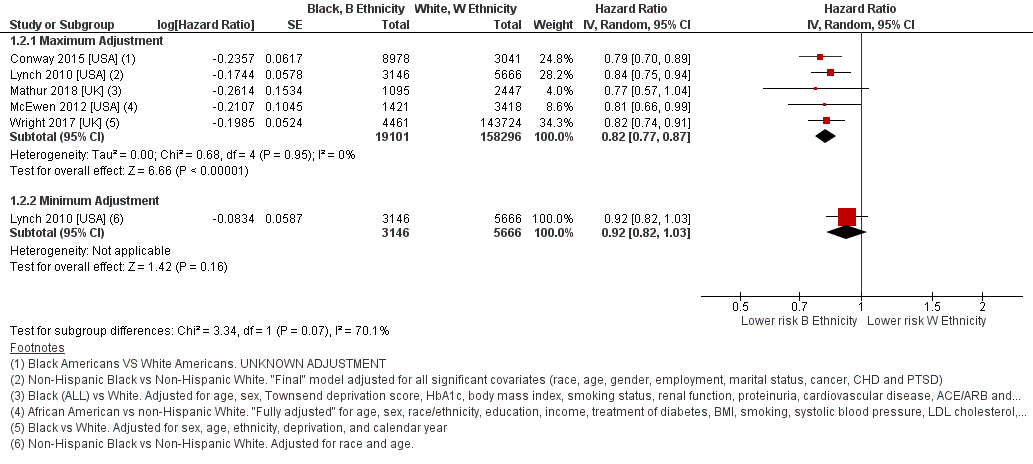
Figure S6 – Minimally versus maximally adjusted models within the Black ethnicity comparisons

Defined ethnic groups within each study in Forest plot footnotes. Adjustment factors also described in footnotes.

B - Black ethnicity. W – White ethnicity.
